# AI performance in oral cytology for differentiating poorly defined tumor cells from reactive atypia

**DOI:** 10.1016/j.jobcr.2025.10.003

**Published:** 2025-10-18

**Authors:** Kaori Oya, Kazuma Kokomoto, Mami Okamoto, Yuko Kondo, Sunao Sato, Kazunori Nozaki, Mitsunobu Kishino, Satoru Toyosawa

**Affiliations:** a Department of Clinical Laboratory, The University of Osaka Dental Hospital, 1-8 Yamadaoka, Suita, Osaka, Japan; b Department of Medical Informatics, The University of Osaka Dental Hospital, 1-8 Yamadaoka, Suita, Osaka, Japan; c Department of Physical Therapy, Takarazuka University of Medical and Health Care, 1 Hanayashikimidorigaoka, Takarazuka, Hyogo, Japan; d Department of Oral and Maxillofacial Pathology, Graduate School of Dentistry, The University of Osaka, 1-8 Yamadaoka, Suita, Osaka, Japan

**Keywords:** Squamous cell carcinoma, Artificial intelligence, Cytodiagnosis, Object detection, Oral cytology, Cell classification

## Abstract

**Background:**

Despite its investigative potential, few studies have reported the use of artificial intelligence (AI) in oral cytology. Oral mucosal cells display significant cellular atypia due to inflammatory stimulation or denaturation, whereas well-differentiated oral squamous cell carcinomas do not always show remarkable cellular atypia. The presence of noncancerous atypical cells alongside ill-defined tumor cells poses significant challenges to the development of effective AI tools. Thus, we aimed to investigate the effect of these atypical cells on AI performance.

**Materials and methods:**

We used 29 cases of non-neoplastic lesions, including gingivitis, stomatitis, and 17 squamous cell carcinomas for supervised learning and validation. The cells were classified into four categories: normal, cancer, orange-suspicious, and green-suspicious. Orange and green suspicious cells indicated tumor cells lacking definitive morphological features. Annotation was performed using VoTT v2.2.0, and YOLOv7 as the object detection model, with model training being performed in six ways.

**Results:**

The model that learned orange- and green-suspicious cells as cancer exhibited the highest detection capabilities, but also yielded a high number of false positives. In contrast, the model that excluded information about suspicious cells could rightfully identify some suspicious cells as cancer with fewer false positives.

**Conclusions:**

Discriminating ill-defined tumor cells from atypical non-neoplastic cells based solely on morphology is challenging. Classifying suspicious cells as cancer often results in numerous false positives. Conversely, AI trained on normal and cancer can reveal previously unnoticed cancerous features in suspicious cells.

## 1 Introduction

Generally, it is easy to make a cytological diagnosis of oral squamous cell carcinoma (SCC) in cases that show clear cytological atypia, such as abnormal variations in nuclear size or shape, cell size or shape, increased nuclear/cytoplasmic ratio, increased number and size of nucleoli, and hyperchromasia. However, diagnosis is sometimes difficult for the reasons stated below. Oral mucosal cells may exhibit significant cellular atypia, such as nuclear enlargement, increased nucleolar size, and hyperchromatic nuclei, due to inflammatory stimulation or denaturation. In contrast, well-differentiated oral SCC may sometimes not present remarkable cellular atypia.^1.2^ Their deceptive morphology results in both over-diagnosis and under-diagnosis, with false-negative rates reported to be approximately 20 %.^1^^,^^2^

Thus, we explored using artificial intelligence (AI) to objectively evaluate subtle morphological changes in oral mucosal cells. The success of AI in various medical image analyses suggests its potential for this application. Cytological images can be processed with a slide scanner, thereby transforming them into digital data. Several AI systems for cytological diagnoses have been documented in gynecology.^3^ However, there is no widely adopted practical AI for oral cytology, although various methods for creating AI are being considered.^4^, ^5^, ^6^, ^7^, ^8^ The presence of both ill-defined tumor cells and reactive atypia may complicate the development process.

Therefore, this study aimed to investigate how these cells influence AI performance and how AI judges these cells using six model training methods to devise an improved training method.

## 2 Materials and methods

### 2.1 Ethical clearance

This study was approved by the appropriate ethical review board. Patients were informed of the opportunity to opt out on the website of our institution. Informed consent was not obtained.

### 2.2 Sample preparation

In total, we used 29 cases of non-neoplastic lesions and 17 cases of SCC. Liquid-based cytology (LBC) specimens of gingival lesions harvested by scraping were used in all cases of this study.

We acquired 28 cases of non-neoplastic lesions (cytological diagnosis: negative for intraepithelial lesion or malignancy [NILM]) including gingivitis, stomatitis, and 13 cases of SCC (cytological diagnosis: 11 of SCC and 2 of oral low-grade squamous intraepithelial lesion or low-grade dysplasia [OLSIL]) for supervised learning and internal validation. In addition, 1 case of non-neoplastic inflammatory lesion (cytological diagnosis: indefinite for neoplasia [IFN]) and 4 cases of SCC (cytological diagnosis: 2 of SCC, 1 of LSIL and NILM each) were prepared for the additional test. The diagnosis of SCC was verified using biopsy, and the non-neoplastic lesion was confirmed either using biopsy (13 cases) or follow-up (15 cases). The SCC cases showed little dysplasia. Both LBC samples and the corresponding paraffin sections of SCC were used for immunohistochemical staining to confirm their identical staining properties.

### 2.3 Image data preparation

**Fig. 1:**
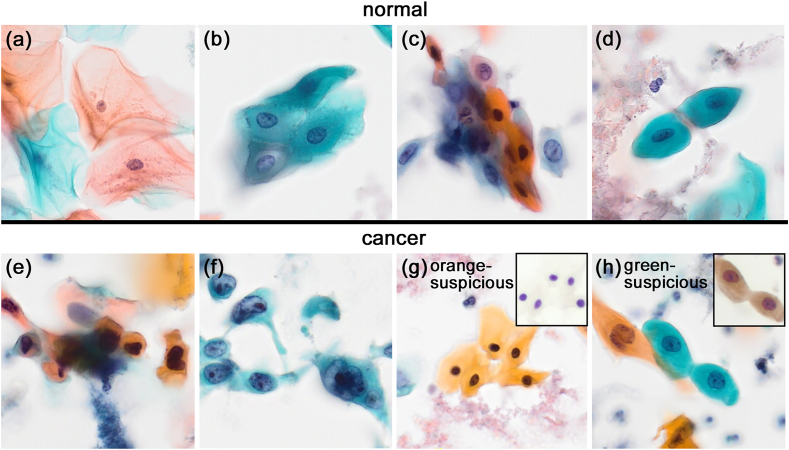
Representative images of “normal” (a–d), “cancer” (e–f), “orange suspicious” (g), and “green suspicious” (h) cells. Immunostaining results of CK13-negative (g, inset) and TUBB3-positive (h, inset) cells are used as supportive information on their cancerous characteristics. The oral mucosal cells display cellular atypia from inflammatory stimulation (c, d). The changes make it difficult to distinguish between atypical normal cells (c, d) and tumor cells with mild atypia (g, h). Original magnification: × 20.

Images were obtained from Papanicolaou-stained LBC specimens of gingival scrapings captured using a slide scanner (OLYMPUS SLIDEVIEW VS200, 40 × ; Evident Corporation, Tokyo, Japan). The cells were classified into four categories: normal, cancer, orange-suspicious, and green-suspicious. Annotations were performed using VoTT v2.2.0.^9^ “Normal” represents non-neoplastic cells; cells were considered “normal” if they were harvested from non-neoplastic lesions, even if they showed atypical features (Fig. 1a–d). “Cancer,” “orange-suspicious,” and “green-suspicious” indicate tumor cells sampled from oral SCC. “Cancer” represents cells with clear cytological atypia as SCC (Fig. 1e and f), which was defined as meeting four or more of the following characteristics or having three or fewer but significant characteristics: abnormal variations in nuclear size or shape, cell size or shape, increased nuclear/cytoplasmic (N/C) ratio, increased number and size of nucleoli, and hyperchromasia. “Orange-suspicious” and “green-suspicious” denote cells with immunostaining-confirmed cancerous properties but lacking definitive morphological features, specifically orange G- and light green-stained atypical cells, respectively (Fig. 1g and h). We separated the two suspicious categories because of the distinct differences in their morphological features. Orange-suspicious shows bright orange cytoplasm and pyknotic nucleus. In contrast, green-suspicious has a rounded, light green cytoplasm and round nucleus with slight swelling. Since there were very few atypical cells with a predominantly eosin Y color, the color classification was not used. One oral pathologist and two cytotechnologists performed the annotations, and plural cells were annotated when multiple cells were present in one image. The annotations were thoroughly reconciled in advance to ensure that they were not subjective and that they did not vary. Of the 5,085 images, 8,242 normal, 2,258 cancer, 255 orange suspicious, and 330 green suspicious labels were created for supervised learning and internal validation. These labels included little or no background. In addition, 32 tumor cell images with clear cellular atypia from 2 SCC cases, including approximately 100 “cancer” (cytological diagnosis: SCC), 64 tumor cell images with ill-defined cellular atypia from 2 SCC cases, including approximately 15 “orange-suspicious” and 20 “green-suspicious” (cytological diagnosis: LSIL and NILM), and 170 non-neoplastic cell images with cytological atypia due to long-term inflammation from one case (cytological diagnosis: IFN), were prepared for the detection test.

### 2.4 Immunohistochemical staining

After scanning the LBC samples by Papanicolaou staining, and immunohistochemical staining was performed using the hypersensitive polymeric method in SCC cases. In each case, one or both of the following primary antibodies were used: rabbit anti-class III beta-tubulin (TUBB3) polyclonal antibodies (1:500, clone D65A4; Cell Signaling, MA, USA) and rabbit anti-cytokeratin (CK) 13 (1:200, clone KS-1A3; Novocastra, Newcastle, UK). Only TUBB3 immunostaining was performed when the specimen did not contain orange G-stained cells. Both TUBB3 and CK13 immunostaining were performed in sequence in cases where the specimen included orange G-stained cells of superficial layer to light green-stained cells of intermediate or deep layer. Paraffin sections from each corresponding case were stained with the same antibodies to confirm the appropriateness of the staining.

TUBB3 and CK13 immunostaining were used to define suspicious cells objectively. TUBB3 expression has been observed in SCC but not in normal stratified squamous epithelium.^10^^,^^11^ The positive rate in tongue and gingival SCC was 91.6 %, and all gingival SCC exhibited TUBB3 positivity in a previous study. In well-differentiated SCC, light-green-stained cancerous cells derived from the intermediate layer express TUBB3 whereas orange G-stained cancerous cells do not.^10^ We used TUBB3 as a positive marker for green-suspicious cells. By contrast, CK13 is a known marker of normal stratified squamous epithelium, and its expression decreases with neoplastic transformation.^12^^,^^13^ Negative expression can be used as evidence of orange- or green-suspicious cells. Yamashina et al. reported that most orange G-stained cells sampled from SCC were negative for CK13 (99.4 %).^12^ Kobayashi et al. showed that all normal/epithelial hyperplasia showed CK13 positive whereas CK13 were not confirmed in all parabasal and lower prickle layers of SCC.^13^ CK13 positive cells were 11.5 % in the upper prickle layer of SCC in their study,^14^ but we considered these to be negligible because we focused on CK13 negative cells in this study.

### 2.5 YOLOv7 training and evaluation

YOLOv7^15^ was used as the object detection model. The default parameters were used to train YOLOv7 with an image size of 640 × 640 pixels. The data were split into 80 % for training and 20 % for internal validation to ensure an even distribution across each class. The performance of the model was evaluated using the mean average precision at an intersection over the union of 0.5 (mAP_50_).

**Fig. 2:**
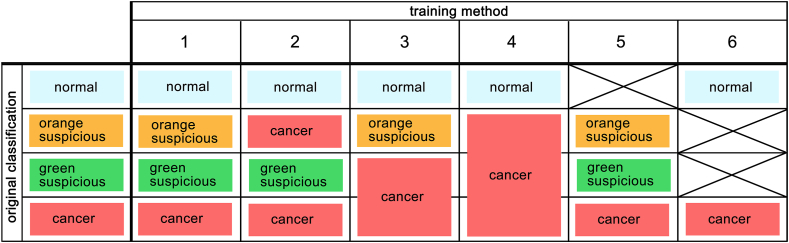
Method of data usage for model training; model training is conducted in the following six ways. 1) four types of cells as per the annotations; 2) three types of cells adding orange-suspicious to cancer; 3) three types of cells adding green-suspicious to cancer; 4) two types of cells adding both orange- and green-suspicious to cancer; 5) three types of cells excluding normal; and 6) two types of cells excluding orange- and green-suspicious.

Model training was conducted using the following six methods to gradually change the use and evaluation of the cell images. The methods were as follows: 1) four types of cells as per the annotations; 2) three types of cells adding orange-suspicious to cancer; 3) three types of cells adding green-suspicious to cancer; 4) two types of cells adding both orange- and green-suspicious to cancer; 5) three types of cells excluding normal; and 6) two types of cells excluding orange- and green-suspicious (Fig. 2).

A detection test was conducted using six models with the prepared images.

## 3 Results

### 3.1 mAP_50_

**Fig. 3:**
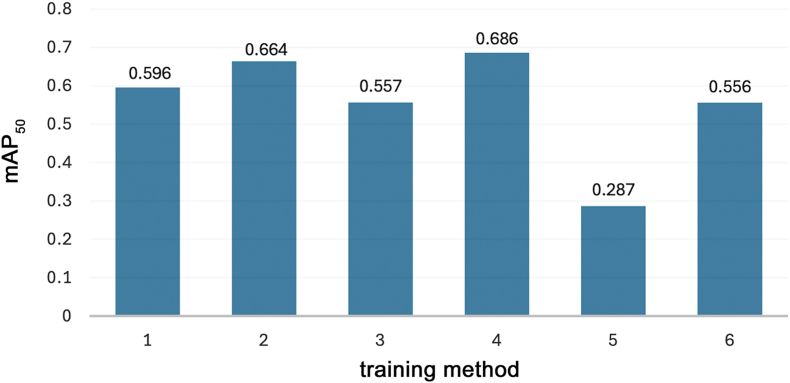
mAP_50_ for each model was calculated. Model 4 shows the highest detection capability, followed by models 2, 1, and 3. Model 5 shows the lowest performance.

Model training was performed in six ways, and the mAP_50_ for each method was calculated (Fig. 3). Model 4, which used both orange- and green-suspicious cells as cancer, showed the highest detection capability (0.686), followed by models 2 (0.664), 1 (0.596), 3 (0.557), 6 (0.556), and 5 (0.287).

### 3.2 Detection test

The number of AI decisions for the additional test images was counted for each model (Table 1a–c).

**Table 1:** Results of the detection test for (a) tumor cell images with clear cellular atypia, (b) tumor cell images with ill-defined cellular atypia, and (c) non-neoplastic cell images with cytological atypia due to prolonged, continued inflammation.

	Training method					
(a)	**1**	**2**	**3**	**4**	**5**	**6**

Normal	24	77	53	53	–	66
Orange-suspicious	0	(cancer)	0	(cancer)	0	–
Green-suspicious	13	11	(cancer)	(cancer)	5	–
Cancer	109	79	88	98	22	59

(b)	**1**	**2**	**3**	**4**	**5**	**6**

Normal	79	181	149	155	–	59
Orange-suspicious	0	(cancer)	3	(cancer)	0	–
Green-suspicious	24	24	(cancer)	(cancer)	6	–
Cancer	33	10	44	40	1	17

(c)	**1**	**2**	**3**	**4**	**5**	**6**

Normal	287	474	429	391	–	453
Orange-suspicious	1	(cancer)	0	(cancer)	0	–
Green-suspicious	45	15	(cancer)	(cancer)	19	–
Cancer	13	0	45	40	6	4

**Fig. 4:**
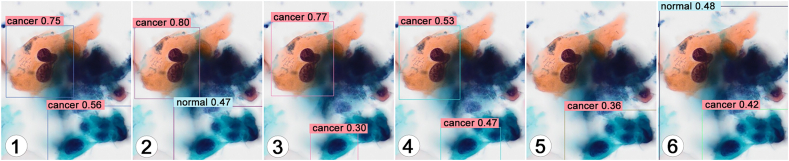
Representative images of the results of the detection test for tumor cell images with clear cellular atypia. Left to right: model 1 to model 6. The classification by AI and its prediction value is shown. The cancer cell detection performance was stable in models 1, 3, and 4. In models 2 and 6, some cancer cells were mistakenly judged as normal (false negatives). In model 5, many tumor cells were not detected. The predictive value tended to decline in model 4 compared to models 1, 2, and 3. Original magnification: × 20. AI, artificial intelligence; SCC, squamous cell carcinoma.

For tumor cell images with clear cellular atypia, the tumor cell detection performance was stable in models 1, 3, and 4 (Table 1a). These models detected cancer almost with high accuracy. Differences in the detected cell count were due to duplicate counts or omissions of the same images. No false-positive results were observed in the detection process. However, in models 2 and 6, some tumor cells were incorrectly classified as normal, which resulted in false negatives (Table 1a, Fig. 4). In model 5, the detected cell number was significantly lower than that in the other models, although there was no misjudgment. The predictive value for cancer tended to decline in model 4 compared to models 1, 2, and 3 (Fig. 4).

**Fig. 5:**
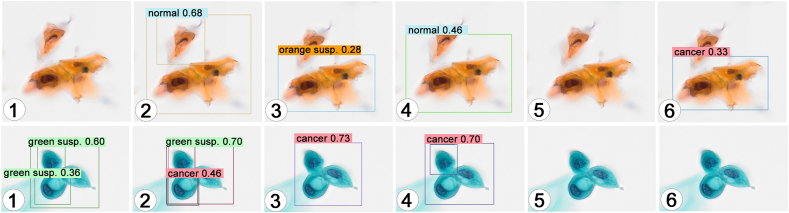
Representative images of the results of the detection test for tumor cell images with ill-defined cellular atypia (orange- and green-suspicious cells). Left to right: model 1 to model 6. Classification by AI and its prediction value is shown. Model 1 detected green-suspicious exactly. Model 2 also detected green-suspicious almost exactly but judged some green-suspicious as cancer (correct interpretation). Many false negatives detected orange-suspicious as normal. Model 3 and 4 judged green-suspicious as cancer as learnt. Model 5 did not judge these images. Model 6 detected some suspicious cells as cancer. Original magnification: × 20. AI, artificial intelligence; SCC, squamous cell carcinoma.

In the detection test for tumor cell images with ill-defined cellular atypia, models 1 and 2 detected green-suspicious almost exactly; however, many false negatives detected orange-suspicious as normal (Table 1b, Fig. 5). Only model 3 detected three orange-suspicious. The “cancer” decisions, which was the correct interpretation, were made more often than expected in all models (Table 1b). The cell images detected as cancer in model 6 showed cellular atypia somewhat closer to cancer than suspicious.

**Fig. 6:**
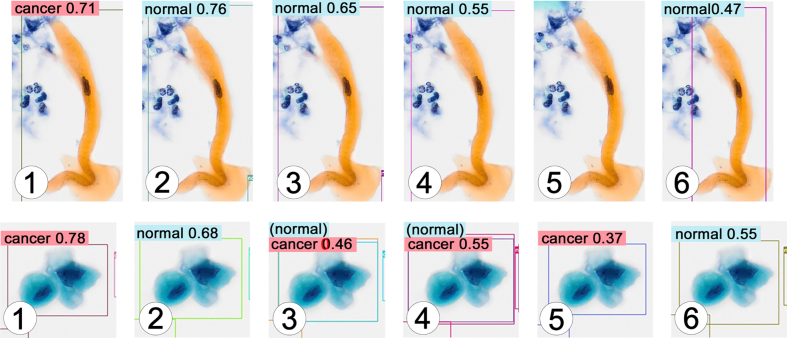
Representative images of the results of the detection test for non-neoplastic cell images with cytological atypia due to long-term inflammation. Left to right: model 1 to model 6. The classification by AI and its prediction value is shown. Model 1, 3, 4 and 5 partially recognized normal cells as cancer (false positives). Model 3 and 4 made two different decisions on one atypical cell: “normal” and “cancer.” Original magnification: × 20. AI, artificial intelligence.

**Fig. 7:**
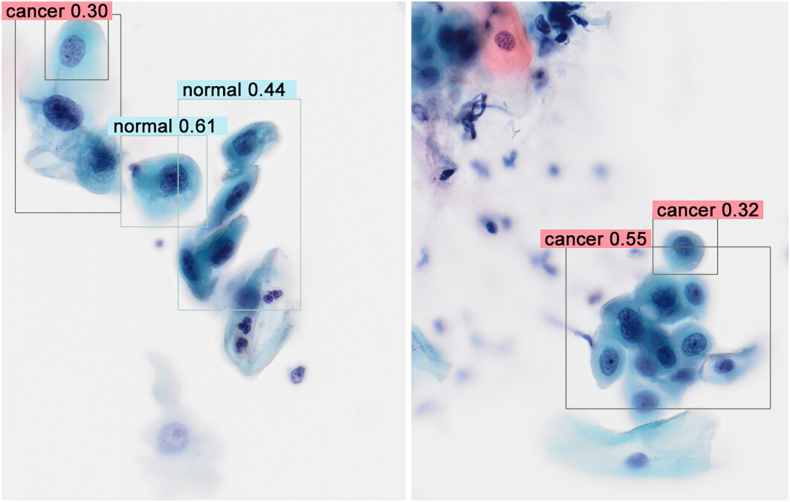
Additional images of the results of the detection test in model 4 for non-neoplastic cell images with cytological atypia. There were many light green-stained cells with enlarged nuclei, increased N/C ratio and hyperchromasia. Many of them detected as cancer (false positives), whereas it is unclear what difference there is between the images judged as normal and those judged as cancer. Original magnification: × 20. N/C, nuclear/cytoplasmic.

For non-neoplastic cell images with cytological atypia, each model, except model 2, partially recognized normal cells as cancer (false positives) (Table 1c, Fig. 6). The number of false positives was the highest in model 3, followed by models 4 and 1 (Table 1c). Light green-stained cells with enlarged nuclei, increased N/C ratio, and hyperchromasia were detected as cancer (Fig. 7). However, it is unclear what difference there is between the images judged as normal and those judged as cancer. (Fig. 7). In model 6, there were a small number of false positives, but the ratio of false positives to true negatives (normal) was very low (Table 1c). The detected cells as false positive were all denatured cells with irregular form.

## 4 Discussion

Since we conducted daily diagnosis with awareness of the four cell types (normal, orange-suspicious, green-suspicious, and cancer), we predicted that model training with the four classifications (model 1) would have the highest score. Moreover, we hypothesized that using information on orange- and green-suspicious cells as cancer (models 2, 3, and 4) would have a negative effect on AI performance because indistinct information causes confusion. To test this hypothesis, model training was conducted using the first to fourth methods (models 1–4). In addition, the fifth and sixth methods (models 5 and 6) were used to discuss the impact of normal and suspicious cell information on AI performance.

Unexpectedly, model 4, which was trained using orange- and green-suspicious cells as cancer, showed the highest mAP_50_, followed by models 2, 1, 3 and 6. It is reasonable that model 5 showed the lowest mAP_50_, because “normal" information may be crucial for the AI to accurately classify other categories, using the difference from normal as a basis for these decisions. Based on the result alone, model 4 appears to be the best. However, considering the results of additional detection tests, AI performance should not be evaluated using mAP_50_ only. For example, model 4 made a lot of false positives. Meanwhile, model 6 reduced false positives and detected overlooked tumor cells although the cancer detections halved. The results suggest that suspicious images may have a part of cancerous features, but it is difficult to distinguish between suspicious and atypical normal by morphology only. In addition, this suggests that we missed the less obvious tumor cells among suspicious images. When we want the AI to determine whether atypical cells are cancer or normal, we should not use information of orange- and green-suspicious. In particular, learning green-suspicious as cancer appears to lead to an increase in false positives. Meanwhile, the impact of the orange-suspicious is not clear. There may be a perception bias of orange-suspicious for AI, contrary to our intentions, because the number of orange-suspicious detected in each model was very small.

These results show that even if the data are the same, the AI performance can vary significantly depending on the training method. Each method used in this study have both advantages and disadvantages. We should choose the training method depending on the purpose. If we allow false positives to detect anything suspicious, Model 4 should be used. When we aim to classify suspicious cells as suspicious, model 1 is the best. Model 6 can be used to identify tumor cells with unnoticed cellular atypia with fewer false positives. If this characteristic can be demonstrated more effectively, it may be possible to discover new cellular characteristics of oral SCC (e.g. structural arrangement) in the future.

However, this study also has some limitations. Firstly, cells were annotated based on numerous characteristics including cytoplasmic color, thickness, size, outline form, chromatin concentration, and nucleoli size and number. Immunostaining evidence was collected, but it is unclear if AI fully captured this complex information. Secondly, the data was sourced from a single institution, thereby potentially introducing bias as staining concentration and color balance can vary between institutions, along with differences in slide scanner settings and conditions. There is also a potential for bias due to the small sample sizes. Lastly, the dataset had an unequal number of “suspicious” images compared to normal and cancer images, which could skew the results despite efforts to balance the classifications. Future research should be expanded to multiple institutions with larger datasets.

## 5 Conclusions

AI performance can vary significantly depending on the training method. Discriminating ill-defined tumor cells from atypical non-neoplastic cells based on cellular morphology alone is difficult. AI identifying suspicious cells as cancerous can yield a high number of false positives. However, AI can recognize cancer characteristics in some ill-defined tumor cells, which we do not recognize, by using the information of “normal” and “cancer” only.

## Patient's/Guardian's consent

The patients were informed of the opportunity to opt out of the study on the website of our institution. Informed consent was not obtained.

## Data statement

The data that support the findings of this study are available from the corresponding author upon reasonable request.

## Ethical clearance

This study was approved by the appropriate ethics review board (R2-E26-1).

## Sources of funding

This study was supported by JSPS KAKENHI Grant Number JP24K15788.

## Declaration of competing interest

The authors declare that they have no known competing financial interests or personal relationships that could have appeared to influence the work reported in this paper.
